# Supplementary material for: Automatic detection of pupil reactions in cataract surgery videos
Source: PLoS One. 2021 Oct 21;16(10):e0258390. doi: 10.1371/journal.pone.0258390 (PMC8530330; doi:10.1371/journal.pone.0258390)
Supplement: S1 Table — (PDF) [file pone.0258390.s001.pdf]

Method A: all types of pupil changes in terms of size and difficulty on thepupil signal on Dataset A

H:

cutoff frequency/ delta	0	0,02	0,04	0,06	0,08	0,1	0,12	0,14	0,16	0,18	0,2
0,01	56,66%	54,51%	52,24%	39,56%	31,45%	30,26%	30,26%	29,68%	26,03%	22,70%	21,46%
0,05	52,57%	57,99%	58,77%	60,94%	56,42%	56,93%	53,19%	49,01%	48,38%	47,88%	48,28%
0,1	31,92%	41,44%	46,28%	50,47%	55,90%	57,83%	59,44%	54,80%	52,31%	50,76%	51,37%
0,15	25,32%	31,83%	37,57%	42,99%	49,01%	53,41%	57,06%	52,52%	51,73%	46,42%	44,48%
0,2	17,86%	25,91%	32,21%	38,69%	44,00%	51,92%	54,76%	49,10%	47,43%	44,44%	40,09%

recall:

cutoff frequency/ delta	0	0,02	0,04	0,06	0,08	0,1	0,12	0,14	0,16	0,18	0,2
0,01	43,01%	39,74%	38,74%	29,81%	22,22%	19,90%	19,90%	19,16%	17,61%	13,67%	12,17%
0,05	73,63%	73,11%	67,51%	64,31%	53,07%	51,61%	47,60%	43,70%	41,16%	40,04%	40,04%
0,1	71,09%	79,81%	77,91%	76,76%	75,36%	69,29%	66,68%	56,49%	48,27%	43,76%	42,55%
0,15	73,76%	73,10%	74,67%	74,47%	73,70%	70,00%	68,20%	59,47%	52,93%	42,69%	38,06%
0,2	65,89%	75,39%	71,13%	74,36%	71,67%	70,50%	67,58%	58,91%	48,75%	42,63%	35,02%

precision:

cutoff frequency/ delta	0	0,02	0,04	0,06	0,08	0,1	0,12	0,14	0,16	0,18	0,2
0,01	75,95%	77,36%	77,36%	64,60%	56,26%	55,57%	55,57%	55,57%	45,57%	44,50%	47,00%
0,05	31,58%	38,09%	40,78%	45,53%	47,85%	50,22%	49,33%	45,83%	47,46%	49,22%	50,50%
0,1	14,57%	20,67%	24,68%	28,67%	34,76%	38,65%	41,78%	41,71%	45,41%	45,77%	48,80%
0,15	10,64%	14,41%	18,15%	22,08%	27,34%	32,51%	37,34%	36,07%	37,74%	36,72%	36,40%
0,2	7,09%	10,93%	14,72%	18,84%	23,05%	30,55%	34,50%	31,46%	32,95%	32,13%	31,45%

GTCR:

cutoff frequency/ delta	0	0,02	0,04	0,06	0,08	0,1	0,12	0,14	0,16	0,18	0,2
0,01	60,50%	59,00%	53,50%	37,29%	30,67%	32,38%	32,38%	32,38%	27,38%	27,38%	27,50%
0,05	84,61%	84,70%	85,36%	85,35%	74,46%	74,61%	66,15%	60,57%	60,10%	57,61%	57,61%
0,1	88,72%	87,16%	87,16%	86,72%	86,02%	86,43%	86,69%	76,55%	68,42%	69,42%	69,42%
0,15	91,23%	89,61%	88,02%	90,48%	90,36%	89,87%	89,87%	79,46%	79,32%	71,57%	73,00%
0,2	84,89%	90,44%	89,59%	90,88%	92,22%	92,13%	90,89%	81,00%	80,67%	77,33%	69,00%

Method B: all types of pupil changes in terms of size and difficulty on the difference signal onDataset A

H:

cutoff frequency/ delta	0	0,02	0,04	0,06	0,08	0,1	0,12	0,14	0,16	0,18	0,2
0,01	60,27%	56,53%	54,48%	44,38%	44,04%	38,50%	38,08%	38,75%	29,83%	25,25%	23,30%
0,05	52,70%	53,74%	53,15%	57,42%	58,88%	59,36%	52,94%	54,76%	54,77%	55,05%	50,78%
0,1	31,65%	36,03%	38,87%	41,23%	43,52%	46,86%	50,39%	50,28%	50,64%	51,50%	52,76%
0,15	24,28%	28,92%	33,25%	36,79%	42,40%	46,54%	48,08%	51,12%	47,28%	45,44%	43,21%
0,2	17,75%	21,14%	24,65%	27,64%	32,72%	37,51%	41,80%	39,41%	42,01%	39,08%	36,34%

recall:

cutoff frequency/ delta	0	0,02	0,04	0,06	0,08	0,1	0,12	0,14	0,16	0,18	0,2
0,01	41,68%	40,18%	38,70%	28,80%	28,40%	26,35%	25,95%	25,95%	19,89%	14,03%	13,23%
0,05	74,29%	70,20%	65,53%	65,50%	64,22%	63,08%	49,27%	48,89%	46,06%	45,30%	42,43%
0,1	71,72%	72,34%	69,39%	67,54%	68,26%	64,97%	64,07%	61,18%	52,24%	47,32%	47,16%
0,15	66,85%	71,40%	72,80%	72,49%	78,40%	75,98%	70,35%	69,24%	60,25%	52,84%	44,51%
0,2	61,27%	63,06%	64,19%	62,94%	63,46%	68,43%	67,26%	58,00%	53,31%	45,52%	39,12%

precision:

cutoff frequency/ delta	0	0,02	0,04	0,06	0,08	0,1	0,12	0,14	0,16	0,18	0,2
0,01	76,41%	75,48%	80,72%	70,21%	70,56%	60,46%	60,39%	65,83%	55,49%	53,06%	47,98%
0,05	31,07%	32,84%	33,24%	38,75%	42,07%	43,41%	42,61%	46,89%	49,71%	51,46%	48,75%
0,1	14,28%	17,14%	19,37%	21,32%	23,08%	26,57%	30,32%	31,11%	35,10%	40,18%	41,57%
0,15	10,26%	12,73%	15,24%	17,60%	21,37%	24,94%	27,00%	30,06%	28,99%	28,21%	28,29%
0,2	7,11%	8,79%	10,67%	12,48%	15,74%	18,40%	21,76%	20,75%	23,77%	23,31%	22,99%

GTCR:

cutoff frequency/ delta	0	0,02	0,04	0,06	0,08	0,1	0,12	0,14	0,16	0,18	0,2
0,01	78,79%	66,95%	59,41%	53,67%	53,38%	42,67%	42,21%	42,21%	30,97%	34,89%	30,92%
0,05	88,60%	89,86%	90,14%	89,44%	86,14%	85,79%	77,46%	76,90%	77,24%	77,00%	66,67%
0,1	92,50%	90,03%	89,67%	90,34%	91,18%	91,03%	91,31%	89,48%	86,07%	81,74%	86,19%
0,15	89,94%	89,58%	91,94%	91,58%	89,25%	89,30%	89,64%	91,03%	80,92%	85,83%	86,14%
0,2	83,21%	81,63%	80,83%	80,11%	80,56%	90,83%	91,32%	93,56%	94,51%	84,09%	74,09%

Method C: obvious pupil changes of any size on the pupil signal on Dataset A

H:

cutoff frequency/ delta	0	0,02	0,04	0,06	0,08	0,1	0,12	0,14	0,16	0,18	0,2
0,01	53,87%	55,47%	51,24%	44,27%	36,07%	34,68%	34,68%	33,71%	28,35%	25,07%	22,70%
0,05	43,14%	51,14%	53,25%	56,42%	53,30%	53,46%	48,37%	48,77%	48,13%	47,28%	47,95%
0,1	21,74%	31,33%	37,81%	42,81%	48,65%	51,45%	48,98%	42,90%	44,37%	44,53%	45,32%
0,15	16,81%	23,49%	28,68%	35,15%	41,48%	44,52%	49,30%	42,82%	40,40%	40,24%	40,27%
0,2	11,94%	17,23%	25,25%	29,57%	35,18%	42,95%	45,12%	38,36%	37,39%	36,93%	37,21%

recall:

cutoff frequency/ delta	0	0,02	0,04	0,06	0,08	0,1	0,12	0,14	0,16	0,18	0,2
0,01	42,03%	42,62%	38,87%	31,93%	24,98%	22,97%	22,97%	21,72%	19,64%	15,70%	13,47%
0,05	77,29%	81,28%	80,03%	75,13%	61,13%	58,43%	51,76%	51,34%	48,15%	45,23%	45,23%
0,1	70,45%	83,70%	86,06%	86,89%	86,30%	77,38%	66,21%	50,73%	48,23%	46,98%	45,31%
0,15	68,28%	81,65%	80,95%	82,62%	83,21%	75,92%	75,09%	60,69%	49,68%	46,59%	42,95%
0,2	62,91%	68,11%	82,90%	80,71%	79,70%	79,29%	72,59%	61,58%	48,02%	44,93%	41,70%

precision:

cutoff frequency/ delta	0	0,02	0,04	0,06	0,08	0,1	0,12	0,14	0,16	0,18	0,2
0,01	56,01%	60,27%	60,61%	58,94%	55,61%	55,00%	55,00%	55,00%	45,00%	44,17%	44,17%
0,05	21,66%	27,88%	29,98%	34,08%	37,57%	38,74%	36,34%	37,45%	38,18%	38,67%	40,05%
0,1	8,79%	13,61%	17,43%	20,73%	25,18%	28,58%	28,85%	28,30%	31,26%	32,06%	34,20%
0,15	6,55%	9,50%	12,19%	15,87%	19,96%	23,35%	27,67%	24,50%	25,32%	26,00%	27,08%
0,2	4,46%	6,74%	10,35%	12,67%	15,97%	21,23%	24,14%	20,31%	22,33%	22,55%	23,61%

GTCR:

cutoff frequency/ delta	0	0,02	0,04	0,06	0,08	0,1	0,12	0,14	0,16	0,18	0,2
0,01	71,21%	71,25%	61,25%	51,35%	39,75%	40,33%	40,33%	40,33%	30,58%	30,00%	28,33%
0,05	95,89%	95,34%	95,34%	95,03%	75,17%	75,86%	65,83%	65,21%	65,06%	64,57%	64,57%
0,1	100,00%	96,67%	96,67%	96,67%	96,59%	96,14%	87,14%	67,14%	67,14%	67,14%	67,14%
0,15	89,17%	97,98%	97,98%	97,98%	98,04%	87,74%	87,74%	78,33%	68,33%	68,33%	70,00%
0,2	90,00%	90,00%	98,75%	98,75%	98,75%	98,75%	88,57%	78,57%	68,33%	68,33%	70,00%

Method D: all types of pupilchanges in terms of size and difficulty on the pupil signal on Dataset B

H:

cutoff frequency/ delta	0	0,02	0,04	0,06	0,08	0,1	0,12	0,14	0,16	0,18	0,2
0,01	44,99%	42,93%	39,74%	35,63%	27,73%	24,64%	19,63%	17,65%	17,44%	17,60%	16,92%
0,05	37,31%	45,17%	47,56%	49,07%	49,32%	48,63%	44,06%	44,53%	35,55%	32,69%	31,40%
0,1	24,80%	28,74%	32,98%	37,43%	43,56%	44,41%	45,33%	45,24%	41,48%	38,05%	32,41%
0,15	17,59%	25,18%	31,98%	34,11%	36,40%	39,83%	41,89%	45,21%	41,69%	40,54%	37,51%
0,2	13,52%	20,43%	26,71%	33,29%	35,69%	35,42%	39,54%	41,42%	36,93%	35,37%	34,82%

recall:

cutoff frequency/ delta	0	0,02	0,04	0,06	0,08	0,1	0,12	0,14	0,16	0,18	0,2
0,01	34,04%	32,58%	28,53%	26,31%	18,35%	16,16%	13,04%	10,06%	9,70%	9,90%	9,48%
0,05	48,42%	52,83%	49,56%	47,00%	41,38%	38,23%	34,81%	33,23%	24,97%	21,09%	19,48%
0,1	47,84%	47,05%	44,13%	44,48%	45,61%	43,02%	41,00%	37,69%	30,44%	26,59%	21,22%
0,15	51,03%	56,48%	56,31%	45,41%	41,52%	41,36%	41,90%	40,71%	32,39%	29,45%	25,95%
0,2	50,40%	54,75%	54,86%	51,90%	43,76%	37,89%	37,00%	35,99%	27,91%	26,40%	24,79%

precision:

cutoff frequency/ delta	0	0,02	0,04	0,06	0,08	0,1	0,12	0,14	0,16	0,18	0,2
0,01	54,76%	59,81%	60,48%	58,81%	52,12%	47,12%	37,50%	37,50%	40,00%	40,00%	40,00%
0,05	21,84%	29,77%	34,54%	38,25%	43,25%	47,35%	44,22%	48,92%	45,02%	42,51%	42,96%
0,1	11,54%	14,57%	18,41%	23,41%	30,44%	34,25%	39,37%	42,89%	45,60%	44,90%	41,67%
0,15	7,19%	11,21%	15,73%	19,10%	22,24%	27,44%	31,61%	39,10%	42,92%	46,02%	44,80%
0,2	5,26%	8,56%	12,11%	16,94%	20,65%	22,49%	28,74%	32,71%	34,56%	36,20%	37,63%

GTCR:

cutoff frequency/ delta	0	0,02	0,04	0,06	0,08	0,1	0,12	0,14	0,16	0,18	0,2
0,01	52,50%	44,50%	41,84%	34,25%	28,98%	25,89%	20,23%	22,80%	22,74%	22,50%	21,32%
0,05	71,68%	71,94%	71,67%	72,96%	73,83%	69,34%	59,69%	59,40%	45,21%	48,01%	47,79%
0,1	74,49%	69,03%	71,47%	66,88%	71,01%	66,19%	61,02%	60,72%	56,97%	52,70%	46,66%
0,15	84,73%	81,45%	80,18%	73,59%	74,78%	67,99%	62,09%	61,63%	56,26%	54,63%	52,34%
0,2	84,99%	85,53%	86,53%	84,58%	78,38%	72,20%	71,16%	71,05%	60,75%	51,80%	51,99%
