# Supplementary material for: Automatic detection of pupil reactions in cataract surgery videos
Source: PLoS One. 2021 Oct 21;16(10):e0258390. doi: 10.1371/journal.pone.0258390 (PMC8530330; doi:10.1371/journal.pone.0258390)
Supplement: Attachment — Submitted filename: Rebuttal Letter (1st revision).docx [file pone.0258390.s002.docx]

# Revision Summary to PLOS One journal (PONE-D-21-15823)

Title: Automatic detection of pupil reactions in cataract surgery videos

Authors: Natalia Sokolova, Klaus Schoeffmann, Mario Taschwer, Stephanie Sarny, Doris Putzgruber-Adamitsch, Yosuf El-Shabrawi

#### Revision Summary

We thank the reviewers for their valuable feedback that helped us to further improve our paper. We have significantly revised our manuscript and tried to resolve all mentioned concerns, as we describe down below. In summary, we have performed the following changes:

- Ethics statement information;
- Improved description of used materials (Sections 2.1 and 2.2);
- Extended evaluation results with the detailed analysis for pupil reactions of different size groups (Section 4.2.3);
- Qualitative subjective study (Section 4.4);
- Extended future work (Conclusion);
- Improved presentation and writing throughout the paper.

To highlight the changes in our current manuscript, we use different colors for the editor and the different reviewers. The colors are assigned as follows: pink for the answers to the editor, green for the responses to reviewer 1, and yellow for the responses to reviewer 2.

### Comments from Editor (E)

#### Comment E-1

###### “Please ensure that your manuscript meets PLOS ONE's style requirements, including those for file naming.”

##### Response

We have updated the submission according to PLOS ONE’s style requirements. The files are renamed in correspondence with the submission rules.

#### Comment E-2 and E-3

###### “Please amend your current ethics statement to include the full name of the ethics committee/institutional review board(s) that approved your specific study. Once you have amended this/these statement(s) in the Methods section of the manuscript, please add the same text to the “Ethics Statement” field of the submission form (via “Edit Submission”).”

###### “Please provide additional details regarding participant consent. In the ethics statement in the Methods and online submission information, please ensure that you have specified (1) whether consent was informed and (2) what type you obtained (for instance, written or verbal, and if verbal, how it was documented and witnessed). If your study included minors, state whether you obtained consent from parents or guardians. If the need for consent was waived by the ethics committee, please include this information. If you are reporting a retrospective study of medical records or archived samples, please ensure that you have discussed whether all data were fully anonymized before you accessed them and/or whether the IRB or ethics committee waived the requirement for informed consent. If patients provided informed written consent to have data from their medical records used in research, please include this information.”

##### Response

We have provided the information regarding the ethics statement and the consent of participants in the text of the paper and the submission system.

##### Revision in the paper

- Page 3, Section 2, lines 99-102

#### Comment E-4

###### “We note that the grant information you provided in the ‘Funding Information’ and ‘Financial Disclosure’ sections do not match.”

##### Response

We have updated the funding information in the submission system.

### Comments from Reviewer 1 (R1)

#### Comment R1-1

###### “Validation of the outcome data is missing, i.e. human observers should be included.”

##### Response

We have performed an additional human observer study and describe the obtained qualitative results in Section 4.4, where we compare the expert opinions with the predictions from our algorithms for five new videos.

##### Revision in the paper

- Page 3, Introduction, line 61
- Pages 10-11, Section 4.4, lines 353-369
- Page 11, Conclusion, lines 390-392

#### Comment R1-2

###### “No sample size calculation is given and a number of 10 and 20, respectively, analysed patients should only be considered as a pre-study analysis.”

##### Response

We have updated the description of the used materials in the paper. We clarify that the used videos were chosen from a very large video dataset (we randomly selected the videos for dataset A from around 600 videos recorded in 2017-2018 and dataset B from around 650 videos recorded in 2019-2020). Since all videos in our study had to be manually annotated for the evaluation of automatic pupil reaction detection, we limited the number of videos to 30 (with 650,890 frames).

##### Revision in the paper

- Page 3, Section 2.1, lines 104-106
- Page 4, Section 2.2, lines 125-127

#### Comment R1-3

###### “Given the low recall and precision values of this algorithm I suggest to re-evaluate the training data set.”

##### Response

We agree with the reviewer that the obtained precision values are rather low, but the achieved recall is reasonably high – which still provides a great benefit for our collaborating clinicians. There is always a compromise between recall and precision: we could configure our method such that recall in combination with precision (i.e., F1 Score) is lowest. However, since this paper aims to find as many pupil reactions as possible, considering even very weak pupil reactions, we used the proposed settings that are optimized for recall, as agreed with clinicians. As was also discussed with clinicians, they need to detect as many reactions as possible, even though some wrong detections might be contained and have to be manually removed from the result set later. Moreover, our algorithm could be used for detecting only strong and medium pupil changes (in terms of intensity) with much better performance.

We have added Section 4.2.3 to show the detailed results of such a configuration (see Table 2 for the results). According to our evaluation, recall of the proposed methodology reaches at least 90% for such significant pupil size changes.

##### Revision in the paper

- Page 2, Introduction, lines 49-50
- Pages 9-10, Section 4.2.3, lines 320-335, 340

#### Comment R1-4

###### “Auto ML deep learning might achieve much better recall and precision values.”

##### Response

We agree with the reviewer that Auto ML deep learning could be used to improve the results of the proposed method.

We tested the AutoML Video Intelligence Classification product from Google (see <https://cloud.google.com/video-intelligence/overview/docs>). We used both datasets (A and B) for model training with the video-wise split of 60:20:20% for training, validation, and test datasets, respectively. We converted our annotations into annotations with two classes: “reaction” and “no_reaction.”

This product allows us to categorize pre-defined segments into two mentioned classes with impressive performance (90.1% precision and 90.6% recall). However, it cannot localize the required segments in the given videos, i.e., it cannot find pupil reactions in a video, though it can classify given segments.

Nonetheless, we would like to apply this approach in future work to improve the predictions of the proposed algorithm. In particular, we plan using AutoML as a post-processing step to improve the performance of our algorithm.

In addition, the AutoML product is not free of charge; thus, we also have to evaluate the financial costs of using such a solution in clinical practice.

##### Revision in the paper

Page 12, Conclusion, lines 393-396

#### Comment R1-5

###### “Recall, precision and GTCR should not be averaged in one value, i.e. harmonic mean, since they are highly divergent in this study.”

##### Response

We agree that recall, precision, and GTCR diverge a lot. We use a harmonic mean of all of them by analogy with the F1 score, which is a harmonic mean of recall and precision, to choose the best configuration. Nevertheless, we analyze recall, precision, and GTCR values independently for the selected configurations in our current version of the manuscript. We added the explanation of that explicitly in the paper in section 4.2.

##### Revision in the paper

Page 8, Section 4.2, lines 282-284

### Comments from Reviewer 2 (R2)

#### Comments R2-1, R2-2, and R2-4

###### “Some parts of the manuscript dont read as scientific as they should. This is particularly true for the introduction and conclusion section. One example: "Clinicians need to operate very precisely to decrease the risk of intra- and postoperative complications to a minimum. Such issues are not only unpleasant for the patient but also costly for hospitals." I suggest that a senior clinican goes carefully through the manuscript and adapts a more scientific approach.”

###### “The conclusion section is summarizing the main results, but lacks the clinical relevance of the approach. Again, a clinician has to take the lead on this part and provide a future persepctive for catarct surgery,”

###### “The paper needs to be corretced by a native speaker. Some of the sentences are very difficult to understand. Examples include "As unclear changes are difficult to detect even with the human eye, we assume that the results should be better if we exclude such pupil size changes from our evaluation." "To keep the numbering of the frames, we insert the missing 176 values with the last satisfying pupil and iris width values." "In this work, we operate with the following terms: pupil, iris, and limbus (a line between the iris and the outer part of the eye)."”

##### Response

We have significantly improved the readability of our paper and added new versions of both the introduction and the conclusion, where we better reflect on the clinical relevance.

##### Revision in the paper

- Page 1, Abstract
- Pages 1-2, Introduction, lines 3-28, 54-55
- Page 4, Section 2.2, lines 132-136
- Page 5, Section 3.1, lines 171-173
- Page 6, Section 3.2, lines 194-198
- Pages 6-7, Section 3.4, lines 214, 225-227
- Page 7, Section 4.1, 246-247
- Pages 11-12, Conclusion, lines 370-389, 396-407

#### Comment R2-3

###### “The authors need to help clinical readers to better understand their approach. Table 1 is given without much explanation on what these different measures (H, Recall, ...) mean.”

##### Response

We extended the description of Table 1 in the current version of the paper.

##### Revision in the paper

Page 9, Section 4.2.2, lines 318
